# Chromatin-Associated RNAs Regulate Gene Expression and Chromatin Structure

**DOI:** 10.3390/ncrna11050068

**Published:** 2025-09-12

**Authors:** Bingning Xie, Ann Dean

**Affiliations:** Laboratory of Cellular and Developmental Biology, National Institute of Diabetes and Digestive and Kidney, Diseases, National Institutes of Health, Bethesda, MD 20892, USA

**Keywords:** chromatin-associated RNAs, *cis* regulation, *trans* regulation, gene expression, chromatin structure

## Abstract

Inside the eukaryotic nucleus, various RNAs are associated with chromatin. These include protein-coding pre-mRNA and different types of non-coding RNAs that are referred to as chromatin-associated RNAs (caRNAs). Recent studies have revealed the important roles of these caRNAs in regulating gene expression and chromatin interactions. In this review, we discuss the recent advances in understanding caRNAs. We first focus on their mode of action, then we summarize the methods used to detect caRNAs and categorize them into three classes: RNA-centric, DNA-centric and protein-centric. Finally, we turn to the proteins that mediate their functions.

## 1. Introduction

Inside of the nucleus the genome is widely transcribed, as more than 75% of the human genome has active transcription to produce protein-coding RNAs and various types of non-coding RNAs [1,2,3]. However, less than 1.5% of the genome encodes for protein-coding genes [4]. Non-coding RNAs inside the nucleus can be divided into two classes based on their length: (1) those shorter than 200 nt are short non-coding RNAs, which include miRNAs, piRNA, snRNAs, snoRNAs and tRNAs; (2) those longer than 200 nt are long non-coding RNAs (lncRNAs), such as Xist and HOTAIR.

A portion of the nuclear RNAs is associated with chromatin, so they are called chromatin-associated RNAs (caRNAs). caRNAs are a class of regulatory RNAs with important functions. caRNAs play various roles, including regulating gene expression and chromatin structure. There are several excellent reviews that comprehensively summarize the functions and mechanisms of caRNAs [1,3,5,6,7]. In this review, we further update the progress in caRNA studies, especially focusing on the non-coding function of pre-mRNA, combinatorial *cis* and *trans* action of caRNAs and advances in the techniques used to study RNA and chromatin interactions.

## 2. Chromatin-Associated RNA Species

### 2.1. Chromatin-Associated Protein-Coding RNAs

In addition to their canonical role in encoding proteins, some protein-coding RNAs also have non-coding functions [8,9,10]. Some protein-coding RNAs have architectural roles in chromatin organization. Chromatin-interlinking RNAs (ciRNAs), a class of long non-polyadenylated coding transcripts, maintain active chromatin architecture, as their depletion disrupts actively transcribed chromatin domains and triggers chromatin condensation [11]. A widespread caRNA is nascent mRNA (pre-mRNA). Beyond studies using perturbation, such as RNase A treatment or RNA polymerase II inhibition, to deplete nascent transcripts globally, individual nascent mRNAs such as *Cpox* pre-mRNA have been shown to regulate chromatin structure and gene expression through non-coding mechanisms [12]. The pre-mRNA transcribed from the *Cpox* locus participates in repressive loop formation by bridging the chromatin interactions between its gene body (containing a silencer-like element) with target neighboring genes and an enhancer locus, exerting a repressive effect on their expression. Knockdown of pre-mRNA of *Cpox* leads to accumulation of PRC2 and H3K27me3 on its genomic locus, which in turn perturbs the chromatin interaction of the *Cpox* gene body genomic locus with target loci, thus activating their expression. However, knockdown of mature *Cpox* mRNA fails to replicate the phenotype, and overexpression of a *Cpox* ORF plasmid (with shRNA-resistant mutations) in *Cpox*-depleted cells does not rescue the defect. This confirms that the observed effect is due to the non-coding function of *Cpox* pre-mRNA rather than its protein-coding role [12]. Emerging evidence from our previous work suggests that the non-coding regulatory function of pre-mRNAs may be preferentially associated with genes located at head-to-tail TAD boundaries [12]. While *Cpox* (a TAD boundary gene) pre-mRNA demonstrates this capacity through chromatin loop formation, it remains unknown whether non-boundary genes can similarly utilize their pre-mRNAs for chromatin regulation. This open question highlights the need for systematic screening of pre-mRNAs across different genomic contexts.

### 2.2. Chromatin-Associated ncRNAs

**Figure 1:**
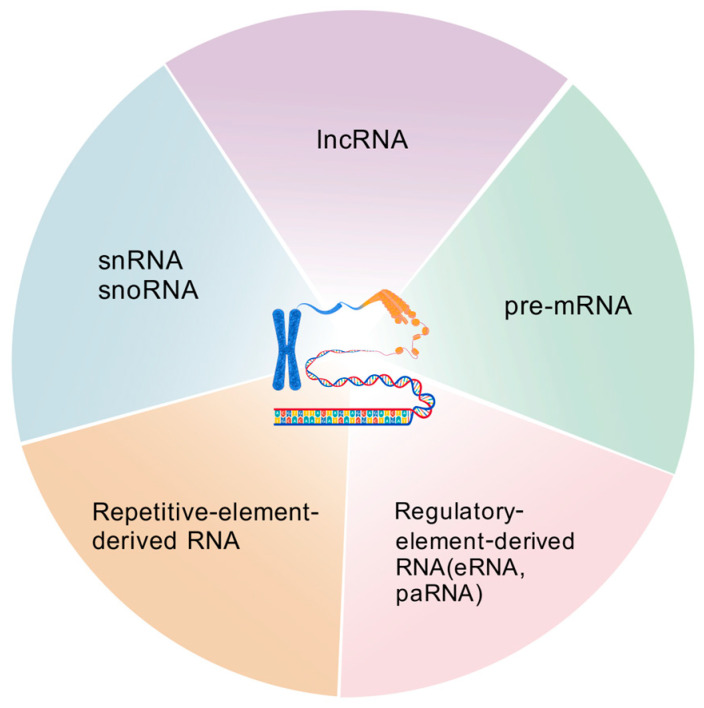
Different categories of chromatin/chromosome-associated RNAs. Figure created with BioGDP.com (accessed on 13 August 2025) [14].

Chromatin-associated ncRNAs can be further divided into several classes, including lncRNAs (with length longer than 200 nt), snRNAs, snoRNAs, regulatory-element-derived ncRNAs (for example, enhancer RNAs (eRNAs) and promoter-associated RNAs (paRNAs)) and repetitive-element-derived ncRNAs [13] (Figure 1).

#### 2.2.1. Chromatin-Associated lncRNAs

lncRNAs are a class of non-coding transcripts with length longer than 200 nt. Some of the lncRNAs interact with chromatin, influencing gene expression and chromatin structure [15,16]. These lncRNAs function either in *cis* (acting near their transcription sites) or in *trans* (regulating distant genomic loci), or via a combination of both modes.

For *Cis*-regulatory functions, they can perform the following functions: (1) Modulate chromatin structure: lncRNAs can interact directly with chromatin, altering its structure to activate or repress gene expression [17]. (2) Recruit chromatin-modifying proteins: Some lncRNAs are localized to specific genomic regions where they can bind and recruit chromatin-modifying proteins (e.g., histones or DNA methyltransferases), thereby regulating gene expression activation or silencing [3,6]. (3) Mediate long-range chromatin interactions: lncRNAs can contribute to the three-dimensional organization of the genome by mediating interactions between distant chromatin regions [18,19]. (4) Perform scaffolding and anchoring: lncRNAs can act as scaffolds or anchors to bring together chromatin-modifying complexes, facilitating their action on chromatin [20].

The *Trans*-regulatory functions include the following: (1) Interacting with other lncRNAs: lncRNAs can interact with other lncRNAs, forming networks that regulate gene expression [21].

(2) Interacting with and regulating genes at distal loci or other chromosomes: lncRNAs can interact with mRNA or protein-coding genes in distal loci or other chromosomes, either directly or indirectly, to regulate their expression and stability [22,23,24]. (3) Affecting other aspects of chromatin biology: lncRNAs can influence DNA replication, DNA repair and the response to DNA damage, as well as other aspects of chromatin biology [25,26].

The in *cis* and in *trans* actions of nuclear RNA are not mutually exclusive, and some caRNAs have been shown to operate in both modes depending on context [6]. A well-known example of a nuclear RNA that acts both in *cis* and in *trans* is Xist. The ncRNAs transcribed from the Xist locus interact in *cis* with the inactive X chromosome (Xi), and this is followed by in *trans* action to spread the repressive histone mark H3K27me3 across the entire Xi [27,28,29].

Collectively, lncRNAs regulate gene expression and chromatin organization through diverse cis-acting and trans-acting mechanisms, with some employing both modes. While their functional versatility is established, unanswered questions include the sequence/structure determinants of their regulatory specificity, the prevalence of functional lncRNAs versus transcriptional noise and how their cis/trans activities are dynamically coordinated.

#### 2.2.2. ncRNAs Derived from Chromatin-Associated Regulatory Elements

Regulatory elements such as enhancers and promoters can be transcribed into non-coding RNAs [30,31,32,33]. The RNA molecules transcribed from enhancers are called enhancer RNAs (eRNAs). eRNAs are a class of important regulatory non-coding RNAs which have variable lengths ranging from 50 to 2000 nt, and most of them are bidirectionally transcribed and non-polyadenylated [31,34,35,36,37,38]. The majority of eRNAs are rapidly degraded by the RNA exosome upon transcription; however, some of them have relatively longer half-lives and have been reported to play roles as RNA molecules [39]. Disruption of the RNA degradation pathway was reported to result in accumulation of eRNAs in the cytoplasm, which indicates that the instability of eRNAs underlies their chromatin enrichment [40,41,42]. The transcription of enhancers and eRNA production is a hallmark of active enhancers, and the expression level of eRNA is positively correlated with enhancer activity [31,36,43,44,45,46,47]. Functionally, eRNAs were reported to mediate chromatin looping between an enhancer locus and target gene locus [48,49] and to promote transcription by establishing chromatin accessibility [50,51]; in addition, eRNAs can also act in *trans* by migrating to a genomic locus on another chromosome [24,48,52,53,54].

A common feature of protein-coding genes is the production of short promoter-associated RNAs (paRNAs) [55,56,57,58,59,60]. These include promoter antisense RNAs (PAS RNAs), which are often expressed in concert with their corresponding sense mRNA transcripts, particularly during differentiation or cellular stress responses [61]. Using a CRISPR-Cas13a- based RNA knockdown strategy and a CRISPR-dCas9- based PAS RNA tethering strategy, PAS RNAs were shown to be functional entities in gene regulation [62]. Mechanistically, PAS RNAs can stabilize the binding of H3K9me3 demethylases KDM4B and KDM4C to ensure removal of the H3K9me3 mark and disrupt the assembly of HP1-7SK-NELFA, thereby facilitating transcription of sense protein-coding RNAs [62].

eRNAs and paRNAs represent functionally important classes of regulatory non-coding RNAs that mediate chromatin interactions, transcriptional activation and epigenetic modifications. While eRNAs contribute to enhancer–promoter communication and chromatin accessibility, paRNAs regulate local transcription through epigenetic complex recruitment. However, key questions remain regarding the structural features determining eRNA stability versus degradation, the mechanisms enabling some eRNAs to act in trans and whether paRNA-mediated regulation represents a widespread or gene-specific phenomenon.

#### 2.2.3. ncRNAs Transcribed from Repetitive DNA Elements

Approximately half of the genome consists of repetitive sequences, like LINE, SINE, LTR, satellites, simple repeats and others. The RNAs transcribed from repeat sequences have important roles in regulating gene expression and chromatin structures. For example, ncRNA transcribed from the human endogenous retrovirus subfamily H (HERVH) interacts with OCT4 and other transcriptional regulators to enhance the expression of neighboring genes and maintain the pluripotency of ESC [63]. Repeat-derived ncRNAs also play roles in facilitating heterochromatin formation. The major satellite repeat RNAs interact with H3K9me3 methyltransferase SUV39H and the nuclear matrix protein SAFB to stabilize heterochromatin architecture [64,65]. Furthermore, repeat-derived RNAs also regulate chromatin compartments, as LINE1 transcript depletion disrupts A/B compartmentalization [66]. LINE1 RNAs and C0t-1 RNAs (a heterogeneous group of repetitive RNA transcripts that are enriched in RNA in situ hybridization when using Cot-1 DNA probes (which target highly repetitive genomic sequences) [67]), maintain the supercoiled chromatin structure through regulating the oligomerization of HNRNPU (SAF-A) [68].

Telomeric-repeat-containing RNA (TERRA) is a long non-coding RNA transcribed from telomeric repeat regions, representing a subclass of satellite RNAs that plays essential roles in telomere function and genome stability. TERRA regulates telomeric chromatin structure, participates in the DNA damage response at telomeres and modulates telomere maintenance by both telomerase and homology-directed repair (HDR). It localizes to chromosome ends via RNA–DNA hybrid (R-loop) formation, a process mediated by RAD51 and RAD51AP1 [69]. These telomeric R-loops can stall replication forks and promote a switch from conventional DNA replication to HDR—a mechanism exploited by telomerase-negative cancer cells that use the alternative lengthening of telomeres (ALT) pathway. In aging cells, TERRA promotes HDR at short telomeres, delaying senescence. Furthermore, during carcinogenesis, elevated TERRA levels can trigger autophagic cell death in response to telomere crisis, highlighting its role as a signaling molecule in telomere-based genome surveillance [70].

Pericentromeric satellite RNAs, transcribed from highly repetitive satellite DNA sequences within constitutive heterochromatin, have emerged as important regulators of nuclear structure and gene expression. Although historically linked to transcriptional silencing, these RNAs are dynamically expressed under specific biological or stress conditions and play diverse roles [71,72,73]. For example, HSat3 lncRNAs serve as scaffolds for nuclear stress bodies (nSBs) during heat stress, sequestering RNA-binding proteins and regulating cellular responses like alternative splicing [71]. Importantly, Pericentromeric satellite RNAs also contribute to the maintenance and repression of pericentromeric heterochromatin via feedback mechanisms [73]. In senescent cells, pericentromeric RNAs can disrupt CTCF binding, leading to increased chromatin accessibility and aberrant activation of senescence-associated secretory phenotype (SASP) genes, potentially driving inflammation and malignant transformation [72]. These findings highlight the context-dependent regulatory potential of pericentromeric RNAs in both normal and pathological states.

caRNAs further drive heterochromatin relaxation. Degradation of heterochromatic transcripts by the Ccr4-Not complex and RNAi pathways is crucial for maintaining heterochromatic silencing. Accumulation of these RNAs on chromatin can lead to the formation of DNA:RNA hybrids, which can negatively regulate heterochromatin assembly and potentially lead to its relaxation [74]. During preimplantation development, pericentromeric-derived caRNAs directly inhibit SUV39H2 histone methyltransferase activity, suppressing H3K9me3 deposition to destabilize heterochromatin condensation [75].

While these studies collectively demonstrate that repetitive sequences and their transcripts play crucial roles in genome architecture and gene expression regulation, fundamental unresolved questions persist regarding (1) how their repetitive nature confers functional specificity, (2) the mechanistic balance between their chromatin-stabilizing and -destabilizing effects.

#### 2.2.4. Chromatin-Associated snRNAs and snoRNAs

Small nuclear RNAs (snRNAs) and small nucleolar RNAs (snoRNAs) are short, functional and the most abundant chromatin-associated ncRNAs in the nucleus. Both snRNAs and snoRNAs mainly function in *trans* due to their longer half-lives and greater mobility [3].

The function of snRNAs is mainly involved in pre-mRNA transcription and processing. For example, 7SK RNA regulates PolII pause–release by controlling the release of p-TEFb [76,77]. U1, U2, U4, U5 and U6 snRNA are components of the spliceosome. U1 snRNP also antagonizes the polyadenylation machinery to prevent premature transcription termination [78,79,80,81].

snoRNAs mainly localize in the nucleolus and participate in the chemical modifications of rRNAs [82]. A subset of snoRNAs was found located in other subnuclear regions, and some of such snoRNAs in mammalian cells that lack modification targets are called orphan snoRNAs. Under DNA damage stress, they show dynamic chromatin binding and function in regulating genome stability and the differentiation of malignant myeloid cells [82,83,84,85].

## 3. Chromosome-Associated RNAs During Mitosis and Meiosis

Mitosis and meiosis represent cellular states characterized by dramatic chromatin and nuclear structural reorganization during which condensed chromosomes exhibit globally reduced transcription. Despite this transcriptional silencing, numerous RNAs remain persistently associated with mitotic/meiotic chromosomes [86,87,88,89,90,91,92]. Quantitative analyses across three human cell lines, a monkey cell line and a mouse cell line revealed on average 20,000 transcripts per cell type maintain mitotic chromosome association, with 5000 demonstrating specific chromosomal enrichment compared to cytosolic levels. Notably, only 821 mitotic chromosome-associated RNAs (mCARs) are conserved across three human cell types, suggesting substantial cell-type specificity that may contribute to cellular identity maintenance. The majority of these mCARs comprise non-coding RNAs, particularly snoRNAs [87]. Shen W. et al. found that highly abundant chromatin-enriched RNAs (cheRNAs), which are predominantly small non-coding RNAs, remain largely associated with mitotic chromatin and represent a major component of chromatin-bound RNA across the cell cycle [86]. The preservation of interphase DNA targeting patterns by mitotic mCARs has led to speculation that these molecules may function as epigenetic memory carriers regulating gene expression [86].

While the precise functions of these retained RNAs remain incompletely understood, several mechanistic insights have emerged. Xist RNA maintains stable transcription and chromatin association throughout mitosis, persistently marking the inactive X chromosome [93]. In Drosophila, SAT III RNA binds kinetochore component CENP-C to ensure proper centromere assembly [94], while mammalian α-satellite RNAs recruit SAF-A during mitotic exit to preserve chromosomal stability [95]. The Ki-67 protein anchors rRNAs to mitotic chromosomes via its FHA domain, facilitating proper chromosomal dispersion during prometaphase [96]. Meiotic examples of retained RNAs include lncRNA R53, which contains a SINE-B1F motif essential for spermatogenesis [90], and *S. pombe* meiRNA that is attached to its genomic locus and mediates chromosomal pairing during meiosis [91,92]. These conserved mechanisms suggest that chromosome-retained RNAs represent a fundamental strategy for cellular memory transmission across cell divisions.

## 4. Methods to Study Chromatin and RNA Interactions

**Figure 2:**
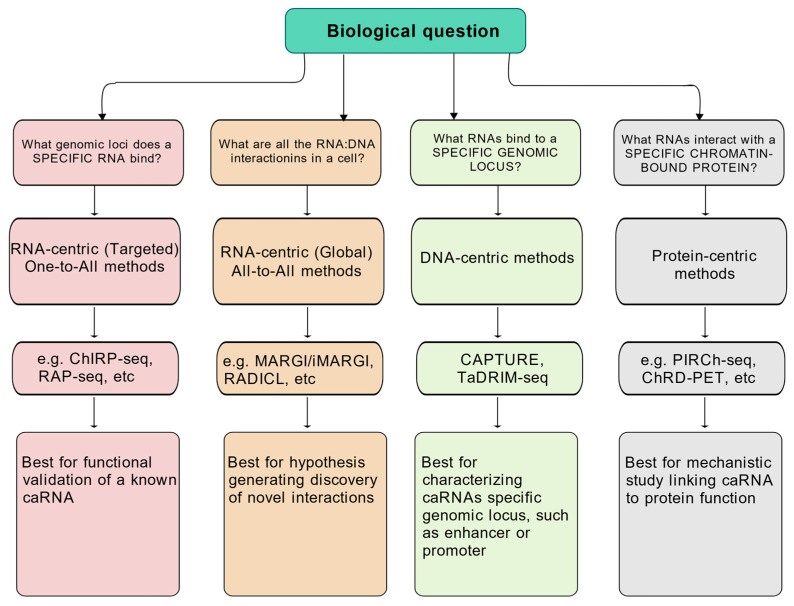
Methods to study chromatin and RNA interactions. Figure created with BioGDP.com (accessed on 20 August 2025) [14].

Since chromatin and RNA interactions often involve proteins, there are generally three ways to study RNA and chromatin association, i.e., RNA-centric, DNA-centric and chromatin-bound protein-centric (Table 1, Figure 2). For the RNA-centric methods, they can be further divided into one-to-all methods and all-to-all methods based on whether the method is focused on a specific RNA or aims to profile caRNAs genome-wide.

### 4.1. RNA-Centric Methods

#### 4.1.1. One-to-All Methods

The one-to-all methods use capture-based approaches with biotin-labeled DNA or RNA probes designed to hybridize with a specific RNA of interest. After pull-down of RNA chromatin complexes and high-throughput sequencing of the associated DNA, the genomic loci interacting with the target RNA can be identified. This kind of one-to-all method can be used to study caRNAs regardless of their expression level [6]. However, because of the intrinsic background noise associated with hybridization-based assays, it is essential to include proper controls, such as RNA knockdown or knockout samples in parallel experiments.

##### Chromatin Isolated by RNA Purification (ChIRP)

ChIRP [97] is one of the earliest and most widely used methods for identifying genomic binding sites of specific RNAs. It uses complementary biotinylated DNA probes to capture target RNAs and their associated chromatin. This method enables high-resolution mapping of RNA occupancy on chromatin. One of its strengths is the ability to distinguish specific interactions by using independently designed probe sets (e.g., odd and even pools), as well as non-targeting control probes. However, ChIRP requires efficient crosslinking and can be sensitive to probe design and RNA abundance. It is more suitable for relatively stable and abundant nuclear RNAs. A limitation is potential nonspecific capture of DNA fragments due to strong crosslinking or hybridization artifacts. ChIRP has been extended to 3D chromatin conformation studies in the HiChIRP method, which combines RNA pull-down with proximity ligation [98].

##### Capture Hybridization Analysis of RNA Targets (CHART)

CHART [99] is another hybridization-based method used to map RNA-associated chromatin regions. Unlike ChIRP, CHART incorporates an RNase H-based step to empirically identify accessible regions on the target RNA for probe hybridization [99], which helps improve specificity and reduce background noise. CHART has been successfully applied to study lncRNAs across various species, including flies [99], lizard [100], mouse [101] and human [102,103]. A key advantage of CHART is its rational probe design based on in situ RNA structure, which minimizes off-target effects. However, CHART may be limited by the availability and efficiency of accessible probe-binding sites, and it generally performs best with well-characterized RNAs with known structures.

##### RNA Antisense Purification (RAP)

RAP [104] is a hybridization-based approach designed to identify genomic binding sites of specific RNAs. It uses long, overlapping probes tiled across the full length of the target RNA, enabling efficient capture even when the RNA is highly structured, partially degraded or bound by proteins. This strategy offers high specificity and comprehensive coverage, making RAP particularly suitable for long and structured RNAs. Compared to ChIRP and CHART, RAP utilizes longer probes, which improves hybridization efficiency but requires more extensive probe synthesis and optimization.

#### 4.1.2. All-to-All Methods

Unlike one-to-all methods, which require prior knowledge of the RNA of interest, all-to-all methods are designed to identify novel caRNAs in an unbiased manner. These genome-wide approaches enable the detection of RNA-DNA interactions without targeting a specific RNA, and several techniques have been developed in recent years.

##### Mapping RNA–Genome Interactions (MARGI) and iMARGI

Sridhar et al. reported a method called MARGI (mapping RNA–genome interactions) [84] which can massively detect native RNA–chromatin interactions from unperturbed cells, enabling detection of genome-wide RNA-binding sites without prior knowledge. This method is based on connecting caRNAs with their target genomic sequences by proximity ligation. Ligation steps are performed on streptavidin beads. The resulting RNA-DNA chimeric sequences were then converted into a sequencing library for paired-end sequencing. Later the same group reported an updated version called iMARGI [105], which differs from the previous version MARGI in that the ligation steps are performed in situ instead of on streptavidin beads. This in situ ligation preserves spatial context and improves interaction detection fidelity. These methods are well suited for investigating the global architecture of RNA–chromatin interactions, although they may have limited resolution and require extensive sequencing depth due to high background.

##### Global RNA Interaction with DNA Sequencing (GRID-seq)

GRID-seq [106] uses a biotinylated bivalent linker to ligate RNA to DNA in situ in formaldehyde and disuccinimidyl glutarate-fixed nuclei. There are several differences in the GRID-seq protocol compared with other techniques: (1) In contrast to the bivalent linker used for MARGI, the linker in GRID-seq is single-stranded RNA with the 5′ end pre-adenylated instead of DNA. (2) The linkers that GRID-seq uses have two restriction sites for a type IIS restriction enzyme (MmeI), thus allowing subsequent digestion to generate size-specific products. Other techniques like ChAR-seq use sonication to achieve fragmentation of ligated products. The advantage of the restriction-based approach in the GRID-seq protocol is that all reads contain RNA and DNA sequences, while in other techniques like MARGI and ChAR-seq, this is not certain. The disadvantage is the decreased mapping power of short reads with GRID-seq [106,107].

##### Chromatin-Associated RNA Sequencing (ChAR-seq)

ChAR-seq [108] is also a proximity-ligation-based method that detects RNA–chromatin interactions. The fragmentation step is performed with sonication, not the restriction enzyme digestion used in GRID-seq [106]. ChAR-seq was first developed for Drosophila cells, then applied to human cells and Xenopus embryos. The disadvantage of ChAR-seq is that it requires more sequencing depths in mammalian cells since random ligation may be more common in mammalian cells than in Drosophila [107].

##### RNA and DNA Interacting Complexes Ligated and Sequenced (RADICL-seq)

RADICL-seq [109] is a proximity-ligation-based technique designed to reduce biases associated with nascent transcription and improve genomic coverage and unique mapping rates. By incorporating transcriptional inhibition with actinomycin D treatment and RNase H treatment prior to crosslinking, RADICL-seq distinguishes more stable RNA–chromatin interactions from transient ones and reduces the bias toward nascent transcription. This method is particularly valuable for studying how transcription shapes chromatin organization, although the additional steps to suppress nascent transcription may reduce the capture of dynamic interactions.

##### RNA Ends on DNA Capture (Red-C)

Red-C [110] identifies RNA–chromatin interactions by capturing the 3′ ends of RNA ligated to fragmented DNA in formaldehyde fixed cells. This method enables the global detection of both coding and non-coding RNA interactions with chromatin. Notably, Red-C revealed that non-coding RNAs associate with both active and repressed chromatin regions, suggesting their broad role in transcriptional regulation and genome organization. However, the reliance on RNA 3′ ends may limit detection of certain RNA species or interaction types.

### 4.2. DNA-Centric Methods

DNA-centric methods aim to identify RNAs associated with specific genomic loci. These methods are especially valuable when investigating low-abundance caRNAs that may not be efficiently detected by genome-wide (all-to-all) approaches.

#### 4.2.1. CRISPR Affinity Purification in Situ of Regulatory Elements (CAPTURE) Approach

To better understand regulation at one genomic locus, one needs to obtain the whole picture of what kinds of RNAs and proteins are bound there. In the case of low-abundance caRNAs, all-to-all methods are less sensitive in detecting them; therefore, all-to-all methods are not suitable for obtaining the whole picture of RNAs bound at a specific genomic locus. To overcome this drawback, Liu X. et al. developed a novel approach called CRISPR affinity purification in situ of regulatory elements (CAPTURE) [111]. This method uses guide RNAs coupled with biotinylated nuclease-dead cas9 (dCas9) to detect locus-specific interactions. After streptavidin affinity purification, RNA-seq was used to detect caRNAs, 3C-seq was used to detect long-range DNA interactions and proteomics was used to detect the *trans*-regulatory factors. Applying this method to the β-globin LCR established evidence for a composition-based hierarchical organization. Spatial features that causally control gene transcription were identified by using this method for unbiased analysis of chromatin interactions at disease-associated *cis*-elements and developmentally regulated super-enhancers. This method is particularly useful for studying low-abundance RNAs at specific loci, which are often missed by global methods. However, it requires careful gRNA design and may be less suited for genome-wide screening.

#### 4.2.2. Targeted DNA-Associated RNA and RNA-RNA Interaction Mapping by Sequencing (TaDRIM-seq)

TaDRIM-seq [112] is a novel technique that combines Protein G (PG)-Tn5-targeted DNA element technology with in situ proximity ligation to capture nearby RNA molecules, thus allowing researchers to simultaneously map both DNA-RNA interactions and RNA-RNA interactions within cells, providing a comprehensive view of the RNA interactome at specific DNA loci. This approach improves upon traditional methods for mapping RNAs associated with specific DNA loci, like ChRD-PET and RedChIP (see below), which are time-consuming, need significant resources and struggle with in vitro ligation efficiency and RNA-RNA interaction detection. TaDRIM-seq is efficient, high-resolution and powerful, while also reducing the need for large cell numbers and time. It can be used in both animal and plant systems.

### 4.3. Protein-Centric Methods

Protein-centric approaches focus on identifying caRNAs that interact with specific chromatin-binding proteins or histone modifications. These methods typically involve immunoprecipitation and allow targeted detection of RNA:DNA interactions mediated or stabilized by protein complexes.

#### 4.3.1. Chromatin-Associated RNA Immunoprecipitation Followed by Next-Generation Sequencing (CARIP-seq)

CARIP-seq [113,114] is a method used to identify and analyze RNAs associated with specific chromatin marks. It modifies ChIP and RIP protocols, basically performing crosslinking with high-concentration (1%) formaldehyde, similar to ChIP, but with a 1 min treatment to avoid over-crosslinking. A reduced number of sonication cycles is used to preserve RNA–protein interactions at condensed heterochromatin regions. Then an antibody targeted to a specific protein of interest is used for immunoprecipitation to pull down RNA and DNA associated with this protein, followed by sequencing. This method is especially useful for exploring RNA associations with repressive chromatin states.

#### 4.3.2. Profiling Interacting RNAs on Chromatin Followed by Deep Sequencing (PIRCh-seq)

PIRCh-seq [115] enables the identification of caRNAs in a histone-modification-specific manner. This method cannot provide information about the exact binding sites of the chromatin-associated lncRNA; thus, it cannot tell whether each lncRNA functions in cis or in trans. PIRCh-seq can better capture RNA–chromatin association by using 1% glutaraldehyde, which forms stronger crosslinks between chromatin and associated RNAs. Compared to other methods, PIRCh-seq reduces contamination from nascent transcripts due to stronger glutaraldehyde crosslinking. However, similar to RIP-seq and CLIP-seq, it may still pull down abundant mRNAs that nonspecifically associate with chromatin or immunoprecipitated complexes. Therefore, to find the bona fide caRNAs, it is necessary to perform further experiments and analysis.

#### 4.3.3. Chromatin-Associated RNA–DNA Interactions, Followed by Paired-End-Tag Sequencing (ChRD-PET)

ChRD-PET (chromatin-associated RNA–DNA interactions followed by paired-end-tag sequencing) [116] combines chromatin immunoprecipitation (ChIP), RNA-DNA proximity ligation and paired-end-tag sequencing (PET) to identify and characterize the specific locations where RNA and DNA interact in the nucleus. Formaldehyde is used to fix the RNA-DNA interactions. The specific RNA-DNA interactions are enriched by immunoprecipitation with an antibody for a target protein or histone modification associated with the RNA or DNA, for example, H3K4me3. Then the DNA fragment and RNA with proximity are ligated and subject to paired-end-tag sequencing to map their interaction sites. ChRD-PET provides paired information and allows identification of interaction loci between RNAs and chromatin with spatial context, thus offering insights into chromatin looping and 3D organization involving RNA.

#### 4.3.4. RedChIP

RedChIP sequencing [117] is a technique used to identify non-coding RNAs associated with specific genomic locations. It combines the principles of ChIP-Seq (chromatin immunoprecipitation sequencing) with RNA-DNA chimera formation to identify and sequence RNA-DNA complexes. Basically, cells are fixed with formaldehyde and DNA fragmentation is achieved by NlaIII digestion. A biotinylated bridge adapter is ligated to RNA 3′ ends, and then the opposite end of the bridges is ligated to the DNA ends in spatial proximity. The ligated complexes are sonicated and immunoprecipitated with an antibody for the target protein. The pull-down complex is subject to reverse transcription, and the cDNA and DNA ends are then used for library preparation and sequencing.

#### 4.3.5. RT & Tag

RT & Tag [118] uses an antibody to capture RNA associated with the chromatin epitope, and then localized reverse transcription generates RNA/cDNA hybrids that are subsequently tagmented by Tn5 transposases. This method enables the mapping of RNA–chromatin interactions, RNA–protein interactions and RNA modifications.

#### 4.3.6. RNA and DNA Interacting Complexes Ligated and Sequenced (RADICL-seq) with Immunoprecipitation (RADIP)

RADIP [119] extends RADICL-seq by combining it with immunoprecipitation to specifically enrich RNA-DNA complexes associated with target proteins; thus, this technique can characterize protein-mediated RNA–chromatin interactions, enhancing its resolution and specificity.

#### 4.3.7. Chromatin Sequencing (Chrom-seq)

Chrom-seq [120] uses a crosslinking-free and antibody-free approach to identify RNA associated with chromatin marks in living cells. This technique combines specific chromatin mark readers with APEX2 enzyme, which catalyzes the oxidation of biotin-aniline to label and isolate the adjacent RNAs by streptavidin-coated beads, allowing the systemic mapping of caRNAs that play regulatory roles in epigenetic events.

**Table 1:** Methods to study chromatin and RNA interactions.

Type		Method	Principle	Strengths	Limitations	Suitable for	Reference
RNA-centric	One to all	ChIRP	Hybridization+ pull-down	High specificity with probe design; compatible with sequencing	Requires strong crosslinking; may miss transient interactions	General Individual RNA- chromatin mapping	[97]
		CHART	Hybridization (guided by RNase H)	Rational probe design reduces background; cross-species applicability	Requires known RNA accessibility; less sensitive for poorly characterized RNAs	Structured RNAs with known access sites	[99]
		RAP	Long, tiled probes	High specificity and coverage; tolerant to RNA structure	Requires complex probe synthesis	long, structured, or degraded RNAs	[104]
	All to all	MARGI/ iMARGI	Proximity ligation	Genome wide and unbiased; no need for RNA specific probes	High background; needs deep sequencing	Global profiling of RNA- chromatin interactions	[84,105]
		GRID-seq	Proximity ligation with linker	All reads contain RNA and DNA; high interaction confidence	short reads may limit resolution	High confidence global interaction mapping	[106]
		ChAR-seq	proximity ligation with sonication	Compatible with complex genomes; longer chimeric fragments	Higher background in mammalian cells	Comparative studies across species; flexible ligation fragment sizes	[108]
		RADICL-seq	Proximity ligation with transcription inhibition	Reduces nascent transcription bias; high mapping efficiency	May miss transient or dynamic interactions	investigating stable RNA-chromatin contacts; transcription-dependent chromatin regulation	[109]
		Red-C	Ligation of RNA 3′ends to fragmented DNA	Capture diverse coding and non-coding RNA interactions	Biased toward RNA 3′end; limited resolution	Mapping broad RNA- chromatin interactions across chromatin states	[110]
DNA-centric		CAPTURE	Locus specific targeting with dCas9-biotin complex	High sensitivity for specific loci; identifies RNA, DNA, and protein interactions	Requires gRNA design; not genome-wide	Detailed analysis of chromatin regulation at specific loci	[111]
		TaDRIM-seq	PG-Tn5 tethering+ in situ proximity ligation	Simultaneous detection of DNA-RNA and RNA-RNA interactions; efficient; low input	Requires targeting design; not genome-wide	Mapping complex RNA networks at specific DNA loci in animal or plant systems	[112]
Chromatin-bound protein-centric		CARIP-seq	ChIP-RIP hybrid: Crosslink +IP of chromatin proteins	Captures RNAs bound to specific chromatin proteins; good for heterochromatin	Limited to protein-specific interactions; resolution depends on antibody quality	Profiling RNAs associated with repressive chromatin marks	[113,114]
		PIRCh-seq	Crosslinking+IP+ caRNA profiling	Histone modification specific caRNA identification; reduced nascent RNA contamination	Cannot identify binding sites; may still co-purify mRNAs	Global profiling of caRNAs	[115]
		ChRD-PET	ChIP+promximity ligation +PET sequencing	Maps specific RNA-DNA interactions with spatial context; reveals RNA-mediated chromatin looping	Technically complex; resolution depends on proximity ligation efficiency	Studying RNA’s role in 3D genome organization	[116]
		RedChIP	ChIP+RNA-DNA chimera formation+sequencing	Identifies ncRNAs associated with protein defined chromatin regions	Resolution limited by proximity ligation and ChIP antibody quality	Studying ncRNAs at specific chromatin environments	[117]
		RT&Tag	Antibody targeting+ in situ RT+Tn5 tagmentation	High resolution, efficient, detects RNA-chromatin/protein/modification	Depends on antibody specificity and reverse transcription efficiency	Mapping RNA-chromatin/ protein/modification in situ	[118]
		RADIP	Crosslinking+RNA-DNA ligation+IP of target protein	High specificity for protein mediated RNA-DNA interactions	Requires good antibody, may miss transient or weak interactions	Studying protein specific RNA-chromatin interactions	[119]
		Chrom-seq	Chromatin mark reader+ APEX2 labeling without crosslinking or antibodies	Live cell, label free mapping of RNAs near specific chromatin marks	Requires efficient expression of fusion proteins; limited by APEX2 labeling radius	In vivo detection of caRNAs at specific epigenetic states	[120]

Note: Blue color shows the RNA-centric methods, red color shows the DNA-centric methods, and green color shows the chromatin-bound-protein-centric methods.

## 5. Non-Canonical Structures in caRNAs

Beyond canonical RNA functions, caRNAs utilize non-canonical structural conformations to mediate precise genomic targeting and epigenetic regulation. These specialized architectures—including G-quadruplexes (G4s), R-loops and RNA:DNA triplexes—enable caRNAs to act as dynamic scaffolds, recruiting effector complexes to specific genomic loci.

### 5.1. RNA G-Quadruplexes (G4s)

**Figure 3:**
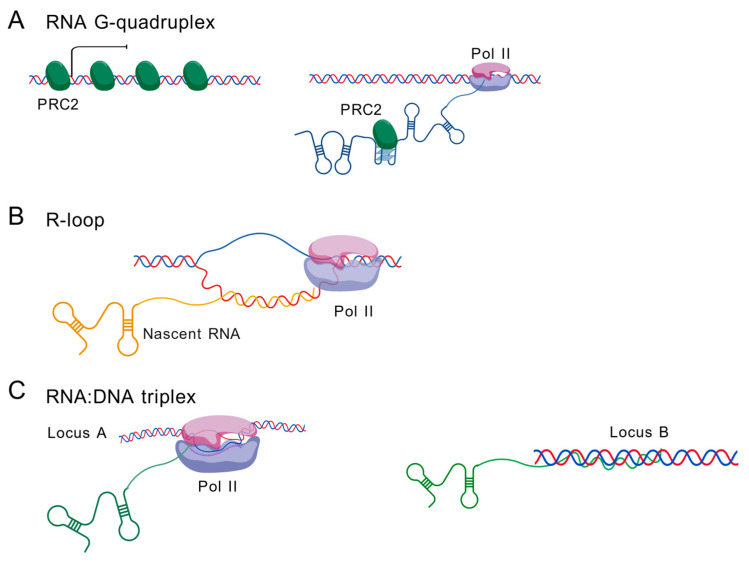
Non-canonical structures of caRNAs. (**A**) RNA G-quadruplex. (**B**) R-loop. (**C**) RNA:DNA triplex. Figure created with BioGDP.com (accessed on 13 August 2025) [14].

RNA G-quadruplexes (G4s) represent a key class of non-canonical RNA structures that plays multifaceted roles in chromatin organization and gene regulation. These stable four-stranded structures form through Hoogsteen base-pairing of guanine-rich (G-tracts) sequences in RNA [121,122]. RNA G4s in caRNAs serve as critical structural and functional elements that regulate chromatin organization, gene expression and RNA processing. At telomeres, the long non-coding RNA TERRA forms G4 structures that act as scaffolds for protein recruitment, facilitating the binding of telomere-protective factors (e.g., TRF2, FUS, EWS) and histone methyltransferases to maintain heterochromatin stability [123,124,125]. Beyond telomeres, RNA G4s influence alternative splicing by recruiting splicing regulators (e.g., hnRNPH/F) to pre-mRNAs, ensuring proper intron removal in transcripts such as p53, PAX9 and BACE1 [126,127,128,129,130]. Additionally, RNA G4 structures function as recognition platforms for chromatin-modifying complexes, particularly PRC2, which exhibits preferential binding to G4-containing RNAs [131]. This PRC2-G4 RNA interaction sequesters PRC2 away from transcriptionally active genes, effectively depleting it from activated loci [132] (Figure 3A). RNA G4s also regulate microRNA biogenesis by inhibiting Dicer processing when G4 structures compete with canonical stem-loops [133,134]. Furthermore, G4s in retrotransposon-derived RNAs may promote transposition, linking them to genome evolution [135]. Collectively, RNA G4s emerge as versatile regulators that integrate RNA structure with chromatin dynamics, impacting transcription, splicing and epigenetic silencing.

### 5.2. R-Loop

R-loops, formed by nascent RNA:DNA hybrids and displaced single-stranded DNA, serve as key mediators of caRNA–chromatin interactions (Figure 3B) [3,6,7]. These structures preferentially arise in GC-skewed regions, repetitive sequences (e.g., telomeres) and highly transcribed loci, where they modulate transcription pausing, termination and antisense RNA synthesis. Notably, R-loops at gene promoters often induce transcriptional pausing, whereas those at the 3′ end can disrupt termination, with transcripts harboring R-loops generally exhibiting higher expression levels [3,7].

Beyond transcriptional regulation, R-loops contribute to chromatin remodeling and epigenetic reprogramming. For example, lncRNAs such as TARID form R-loops at promoter regions (e.g., TCF21), recruiting DNA demethylases (TET1 via GADD45A) to activate gene expression [136]. Similarly, TERRA lncRNA invades telomeric DNA via R-loop formation, mediated by RAD51, to maintain telomere structure—though excessive accumulation leads to fragility [69]. R-loops also facilitate long-range chromatin interactions, as enhancer-derived RNAs (eRNAs) form trans-acting R-loops or triplex structures to bridge enhancer–promoter contacts [137].

Additionally, R-loops collaborate with chromatin remodelers (e.g., BRG1 of the SWI/SNF complex) to resolve transcription–replication conflicts [138], highlighting their role in maintaining genomic stability. The interplay between R-loops and G-quadruplexes (G4s) further amplifies their regulatory impact, as G4 structures on displaced DNA strands stabilize R-loops, linking RNA secondary structure to transcriptional outcomes [139]. Taken together, R-loops serve as dynamic platforms through which caRNAs coordinate gene expression, chromatin accessibility and higher-order genome organization, balancing functional roles with the need to prevent DNA damage.

### 5.3. RNA:DNA Triplex

Although the triplex structure is also an RNA:DNA hybrid structure, triplexes are distinct from R-loops. In the triplex formation process, an RNA molecule establishes sequence-specific interactions by forming Hoogsteen bonds with purine bases in the DNA major groove (Figure 3C) [3,6]. Many caRNAs use this triplex mechanism to act in *trans* to regulate distal genes or genes on other chromosomes. For example, *Khps1* targets the promoter of SPHK1 through a triplex structure [17]. Meg3 forms an RNA:DNA triplex with thousands of target loci across the genome [140]. However, due to lack of techniques to map RNA:DNA triplexes genome-wide, further studies are needed to uncover whether additional RNAs use this mechanism [3].

## 6. caRNA-Interacting Proteins

Studies suggest that caRNAs may facilitate long-range chromatin architecture by interacting with proteins, DNA or other RNAs [3,6,7,21]. RNA is normally present in the cell in the form of ribonucleoprotein (RNP) complexes, and its associated proteins are crucial for both *cis*- and *trans*-regulatory functions. In this section of the review, we primarily focus on the mechanisms of caRNA–protein–chromatin interactions, and therefore do not elaborate in depth on direct RNA–RNA or RNA–DNA interaction modes. Many RNA-binding proteins and non-canonical RBPs have been shown to interact with caRNAs, supporting their localization, stability and function, particularly in *trans*. In this section, we focus on three key chromatin-associated proteins—CTCF, cohesin and PRC2—and discuss their roles in working with RNA to regulate chromatin architecture. Multiple lines of evidence demonstrate that both CTCF and PRC2 can bind RNA. For CTCF, the lncRNA *Jpx* interacts with CTCF to displace it from the Xist promoter during the onset of X chromosome inactivation [22]. The zinc finger domain of CTCF mediates both its self-clustering and RNA recruitment [141,142,143]. Mutating this RNA-binding domain disrupts around 50% of chromatin loops in mESCs and causes gene dysregulation, demonstrating the functional significance of CTCF-RNA interactions [141,142]. For PRC2, while PRC2 exhibits promiscuous RNA-binding capacity [144,145,146], it shows selectivity for specific RNAs such as lncRNA RepA [145] and high affinity for G-quadruplex-containing RNAs [147,148]. However, whether CTCF/PRC2 interact with RNA is under debate. Guo et al. [149] used a denaturing method to purify in vivo crosslinked RNA–protein complexes and concluded that CTCF/PRC2 binding to RNA was an experimental artifact. However, this conclusion may arise from data misanalysis, as the pipeline Guo et al. used inflated background reads and suppressed mappable signals [150].

### 6.1. CTCF

**Figure 4:**
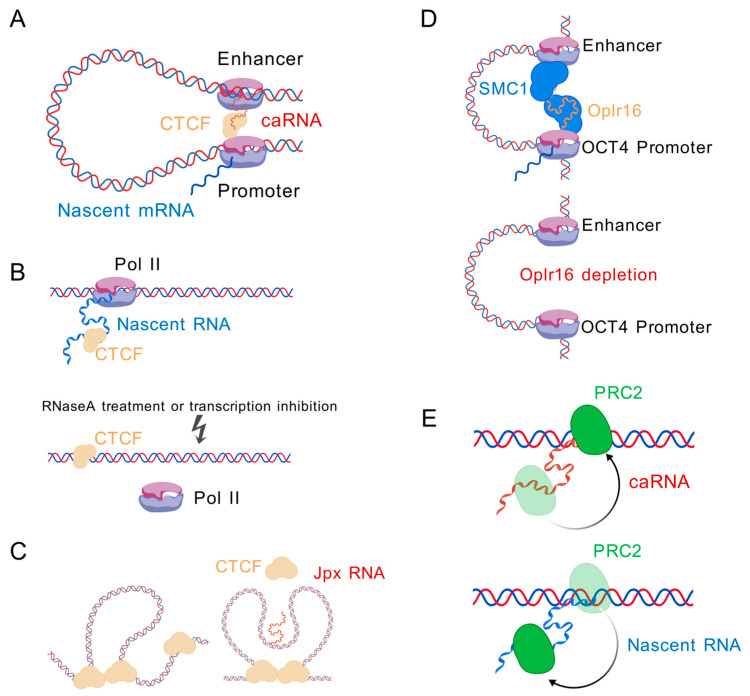
Proteins interacting with caRNAs. (**A**) caRNAs interact with CTCF to facilitate promoter–enhancer loop formation and gene expression. (**B**) Jpx RNA evicts CTCF from chromatin. (**C**) Nascent RNA competes with chromatin for CTCF binding. (**D**) The lncRNA Oplr16 binds to the OCT4 promoter and recruits SMC1 to promote promoter–enhancer looping and OCT4 expression. (**E**) Dual functions of caRNA-PRC2 interaction: recruitment of PRC2 to chromatin (**upper**) and eviction of PRC2 from chromatin (**lower**). Figure created with BioGDP.com (accessed on 20 August 2025) [14].

CTCF is the important architectural protein localized at the anchors of chromatin loops and TAD boundaries [16]. Recent studies have shown that some ncRNAs can facilitate CTCF binding to chromatin to regulate chromatin looping and transcription (Figure 4A) [23,151,152,153,154]. lncRNA MYCNOS promotes CTCF recruitment to the MYCN promoter, driving chromatin remodeling that upregulates MYCN expression and modulates downstream transcriptional programs [153]. lncRNA HOTTIP mediated recruitment of CTCF/cohesin, and R-loop regulators initiated boundary-associated R-loop formation, which in turn stabilized chromatin loops and promoted TAD organization [23]. Boundary-associated RNAs promote the recruitment and spatial clustering of CTCF proteins at TAD borders. This CTCF enrichment subsequently strengthens TAD insulation, modulates enhancer–promoter communication within TADs and fine-tunes domain-wide gene expression patterns [154]. The lncRNA CCAT-L, located upstream of Myc, regulates its expression by facilitating enhancer–promoter looping through two distinct mechanisms. First, it recruits CTCF to modulate the chromatin architecture between the Myc promoter and enhancer [155]. Second, CCAT-L interacts with HNRNPK—often in conjunction with promoter-derived uaRNAs—to promote chromatin looping via HNRNPK oligomerization [21]. An RNA-binding domain mutation of CTCF was reported to disrupt about half of all chromatin loops [141,142,156]. The disrupted loops can be subdivided into two classes depending on whether the CTCF and cohesin binding were lost. The most disrupted loops are mainly enriched in the B compartment, with lost binding of CTCF and cohesin, while the unaffected loops are generally associated with active genes in the A compartment without changes in CTCF and cohesin occupancy [141]. These findings indicate that the interaction between CTCF and RNA is important for chromatin looping.

In contrast to RNAs that recruit CTCF and facilitate its binding to chromatin, global depletion of nascent RNA by transcription inhibition or RNase treatment was reported to increase CTCF binding to chromatin, suggesting that nascent RNA can antagonize CTCF–chromatin interactions (Figure 4B) [157,158]. However, the transcription inhibition and RNA degradation results could not distinguish whether the effect was from loss of pre-mRNA or other types of RNA. Jpx is a non-coding RNA which was initially found to activate Xist expression by evicting CTCF from the Xist promoter [22]. Jpx functions as a CTCF release factor and selectively evicts CTCF bound to low-affinity, developmentally sensitive sites genome-wide (Figure 4C) [159]. Our previous study identified a novel eRNA, *CpoxeRNA* [48], which binds to CTCF/cohesin and antagonize cohesin binding to chromatin. Knockdown of the *CpoxeRNA* leads to accumulation of Rad21 at CTCF-binding sites (CBSs) located at its promoter and interaction targets. However, the chromatin loops anchored by CTCF between the *CpoxeRNA* genomic locus and target loci appear to rely on the eRNA itself, as chromatin looping decreased after *CpoxeRNA* knockdown, although CTCF binding increased.

### 6.2. Cohesin

lncRNAs play roles in facilitating chromatin loops formed by the cohesin complex. For example, Oplr16 (Oct4 promoter-interacting long non-coding RNA 16)—a pluripotency-associated lncRNA—binds specifically to the Oct4 promoter. Depletion of Oplr16 disrupts the chromatin loop between the Oct4 promoter and enhancer, indicating its essential role in maintaining this interaction (Figure 4D). Mechanistically, Oplr16 interacts with cohesin subunit SMC1 via at least four binding sites in its 3′ fragment, thereby recruiting SMC1 to stabilize the Oct4 enhancer–promoter loop and sustain pluripotency [160].

### 6.3. PRC2

PRC2 (polycomb repressive complex 2) is a key chromatin-modifying complex that mediates transcriptional repression by trimethylating histone H3 on lysine 27 (H3K27me3). RNA has been reported to interact with PRC2 and modulate its function, such as regulating its chromatin recruitment, enzymatic activity and target specificity (Figure 4E). One well-known example is the lncRNA Xist, which directly binds to PRC2 and facilitates its recruitment to the inactive X chromosome during X chromosome inactivation [144,145,146,161]. Another example is HOTAIR, an lncRNA transcribed from the HOXC locus, which serves as a scaffold to recruit PRC2 to the targets loci and promote transcriptional silencing [162,163,164]. In addition, nascent RNAs transcribed from active genes have been shown to inhibit PRC2 binding to chromatin, suggesting that RNA binding may prevent PRC2 from accessing active chromatin regions [144,145].

However, recent transcriptome-wide analyses, such as iCLiP, have shown that PRC2 predominantly interacts with nascent, unspliced transcripts from actively transcribed genes [144]. These interactions are largely co-transcriptional and widespread, suggesting a more general role for nascent RNA in modulating PRC2 activity. Nascent RNAs compete with chromatin to bind to PRC2, thus preventing active genes from inappropriate silencing by PRC2. This nascent RNA-PRC2 interaction has an indirect role in regulating neighboring genes and eRNAs, as a consequence of H3K27me3 accumulation at the nascent RNA gene locus upon loss of nascent RNA [12].

## 7. Perspective

Over the past decade, our understanding of caRNAs has expanded from isolated examples of nuclear-retained transcripts to a rich and complex network of RNA species—including nascent mRNAs, diverse classes of non-coding RNAs, regulatory-element-derived transcripts, repeat element RNAs and small nuclear RNAs—that collectively shape genome organization and function. caRNAs play dynamic roles across the cell cycle, with select RNAs like Xist and satellite transcripts persistently remaining bound to mitotic/meiotic chromosomes [93,94,95]. These RNAs maintain cellular memory and ensure chromosome stability and proper dispersion, yet key questions persist about their retention mechanisms during chromatin condensation and their role in epigenetic inheritance. The ability of caRNAs to form non-canonical structures such as G-quadruplexes, R-loops and RNA:DNA triplexes, as well as to engage architectural proteins (e.g., CTCF, cohesin) and chromatin-modifying complexes (e.g., PRC2), underscores their central role in shaping genome architecture and gene regulation. Beyond classical RNA-binding proteins (RBPs) containing canonical RNA-binding domains, accumulating evidence reveals that many DNA/chromatin-associated proteins (e.g., transcription factors CTCF, histone modifiers PRC2) can also bind RNAs [6]. The role of these non-canonical RBPs needs to be further studied to fully understand the mechanims of caRNAs.

Recent technological advances—spanning RNA-centric (e.g., ChIRP [97], RAP [104], RADICL-seq [109]), DNA-centric (e.g., CAPTURE [111], TaDRIM-seq [112]) and protein-centric (e.g., RedChIP [117], RT & Tag [118]) approaches—have enabled increasingly fine-grained maps of RNA–chromatin interactions. However, these methods often operate in isolation, limiting our ability to fully resolve the tripartite RNA–DNA–protein interactome. The integration of these platforms, particularly with single-molecule and single-cell resolution, while capturing structural, positional and temporal dynamics, will be crucial to dissecting functional relationships from mere spatial proximity.

Several pressing challenges define the next phase of caRNA research. First, there is an urgent need for standardized annotation frameworks to distinguish functional caRNAs from pervasive transcriptional noise, especially for repetitive-element-derived species that may be overlooked or misclassified. Second, decoding the structural plasticity of caRNAs—particularly the dynamics of G4s, triplexes and R-loops—in native nuclear contexts may uncover a new regulatory layer in chromatin biology. Third, moving beyond static maps to dynamic, temporal analyses will be essential for understanding how caRNA-mediated interactions respond to developmental cues, stress signals and pathological states.

Looking forward, we envision the convergence of single-cell multi-omics, live-cell imaging and structure-resolved interactome mapping to illuminate the causal links between caRNA localization, structure and transcriptional outcomes. Coupled with computational models that integrate evolutionary conservation, structural motifs and interaction profiles, these approaches could enable the prediction—and even therapeutic targeting—of functional caRNA elements. Such advances will transform caRNAs from enigmatic nuclear byproducts into actionable regulators of chromatin state, with broad implications for developmental biology, genome stability and disease intervention.

## Abbreviations

The following abbreviations are used in this manuscript:

caRNAs	Chromatin-associated RNAs
lncRNAs	Long non-coding RNAs
eRNAs	Enhancer RNAs
paRNAs	Promoter-associated RNAs
circRNAs	Circular RNAs
snRNAs	Small nuclear RNAs
snoRNAs	Small nucleolar RNAs
asRNAs	Antisense RNAs
HERVH	Human endogenous retrovirus subfamily H
ChIRP	Chromatin isolated by RNA purification
CHART	Capture hybridization analysis of RNA targets
RAP	RNA antisense purification
CARIP-seq	Chromatin-associated RNA immunoprecipitation followed by next-generation sequencing
PIRCh-seq	Profiling interacting RNAs on chromatin followed by deep sequencing
MARGI	Mapping RNA–genome interactions
GRID-seq	Global RNA interaction with DNA sequencing
ChAR-seq	Chromatin-associated RNA sequencing
RADICL-seq	RNA and DNA interacting complexes ligated and sequenced
PAS RNAs	Promoter antisense RNAs
Red-C	RNA ends on DNA capture
CAPTURE	CRISPR affinity purification in situ of regulatory elements
TaDRIM-seq	Targeted DNA-associated RNA and RNA-RNA interaction mapping by sequencing
ChRD-PET	Chromatin-associated RNA–DNA interactions, followed by paired-end-tag sequencing
RADIP	RNA and DNA interacting complexes ligated and sequenced (RADICL-seq) with immunoprecipitation
Chrom-seq	Chromatin sequencing
mCARs	Mitotic chromosome-associated RNAs
